# The Influence of Sidedness in Unilateral Cleft Lip and Palate on Mid Facial Growth at Five Years of Age

**DOI:** 10.1177/10556656241233220

**Published:** 2024-02-12

**Authors:** Matthew Fell, David Chong, Paras Parmar, Ting-Li Su, Lars Enocson, Bruce Richard

**Affiliations:** 1 Spires Cleft Centre, Oxford, UK; 2 The Cleft Collective, Bristol Dental School, University of Bristol, Bristol, UK; 3 The Royal Children's Hospital, Melbourne, Australia; 4 156747 Worcestershire Royal Hospital, Worcester, UK; 5 Lancashire Clinical Trial Unit, University of Central Lancashire, School of Medical Sciences, University of Manchester, Preston, UK; 6 Birmingham Women and Children's Hospital, Birmingham, UK

**Keywords:** cleft lip and palate, facial growth, midfacial growth

## Abstract

**Objective:**

To determine whether facial growth at five years is different for children with a left versus right sided cleft lip and palate.

**Design:**

Retrospective cohort study.

**Setting:**

Seven UK regional cleft centres.

**Patients:**

Patients born between 2000-2014 with a complete unilateral cleft lip and palate (UCLP).

**Main Outcomes Measure:**

5-Year-Old's Index scores.

**Results:**

378 children were included. 256 (68%) had a left sided UCLP and 122 (32%) had a right sided UCLP. 5-Year-Old's index scores ranged from 1 (good) to 5 (poor). There was a higher proportion of patients getting good scores (1 and 2) in left UCLP (43%) compared to right UCLP (37%) but there was weak evidence for a difference (Adjusted summary odds ratio 1.27, 95% CI 0.87 to 1.87; *P* = .22).

**Conclusions:**

Whilst maxillary growth may be different for left versus right sided UCLP, definitive analysis requires older growth indices and arch forms.

## Background

There is directional asymmetry in the sidedness of unilateral cleft lip and palate (UCLP), with left sided UCLP twice as prevalent as right sided UCLP.^1^ Anatomical differences have been noted in the soft tissues of the face when orofacial cleft cohorts are stratified by sidedness; right sided unilateral cleft lip with or without palate (UCL/±P) has been associated with a greater degree of hypoplasia of the lateral lip element,^2^ more pronounced facial disfigurement^3,4^ and facial asymmetry^5^ compared to left sided UCL ± P. As these differences have been observed in the soft tissues of the face between left and right sided clefts, it is plausible to consider that there could be differences in the underlying bony structures of the facial skeleton.

Maxillary growth is an important outcome in patients born with UCLP due to the risk of maxillary hypoplasia and subsequent retrusion, which results in malocclusion and malalignment of the jaws, lips and tongue.^6,7^ Most maxillary growth occurs in the first eight years of life from ossification centres located in the transverse palatal suture, giving rise to anterior-posterior growth and the mid-palatal suture, giving rise to transverse growth.^8,9^ Many studies have focused on the impact of extrinsic factors on midfacial growth and it is widely accepted that surgical intervention on the palate can harm maxillary growth plates.^9–13^ Furthermore, reports from rare cohorts of patients with unrepaired orofacial clefts have demonstrated intrinsic growth deficiency compared to controls.^10,14^ Less is known about the impact of intrinsic factors for maxillary growth such as cleft phenotype and sex.

The aim of this study is to investigate the relationship between the sidedness of UCLP and facial growth at five years of age.

## Methods

### Registration

This study was nested within a larger ongoing investigation of midfacial growth in children born with complete UCLP. The purpose of the larger study is to investigate intrinsic factors leading to poor mid facial growth via the analysis of pre-surgical 3D scans of the maxillary arch. Ethical application assessed by the North West – Greater Manchester Central Research Ethics Committee (REC Ref: 18/NW/0536, IRAS: 225282, Research Registry unique identification number: 4068). Health Research Authority (HRA) and Health and Care Research Wales (HCRW) approval was given to this study.

### Participants and Exposure

Children were included if they were born in the United Kingdom (UK) between 2000-2014 with a complete UCLP and receiving treatment from seven UK regional cleft centres: West Midlands, North Thames, South West, Spires, North West, Scotland, Northern and Yorkshire (See Table 1). For inclusion, children needed dental models to have been taken in infancy prior to primary surgical intervention and at the age of five years. The exposure variable was UCLP sidedness, and this was determined by two authors (L.E., B.R.) from 3-D scans of dental models of infants prior to their first surgical procedure. Patient records were used to confirm the complete UCLP cleft subtype and to identify the co-variable of patient sex.

**Table 1.** UK Regional Cleft Centres Involved in this Study and the UK Surgical Pathway Utilised for the Primary Surgical Management of Children Born with a Complete Unilateral Cleft Lip and Palate.

UK Cleft Centre	Surgical sites	Number of cleft surgeons	UK cleft surgery pathway for cleft lip and palate
West Midlands	Birmingham Children's Hospital (Birmingham)	3	Cleft lip reconstruction and vomer flap for hard palate reconstruction at 3-6 months. Intravelar veloplasty for soft palate reconstruction at 6-12 months
North Thames	Great Ormond Street Hospital for Children (London) and St Andrew's Centre, Broomfield Hospital (Chelmsford)	3	
South West	Bristol Royal Hospital for Children (Bristol) and Morriston Hospital, South Wales	3	
Spires	John Radcliffe Hospital (Oxford) and Salisbury District Hospital (Salisbury)	3	
North West	Royal Manchester Children's Hospital (Manchester) and Alder Hey Hospital (Liverpool)	4	
Scotland	Royal Hospitals for Children (Glasgow and Edinburgh)	3	
North East	Royal Victoria Infirmary (Newcastle) and Leeds General Infirmary (Leeds)	3	

### Outcome Measure

The outcome variable was maxillary growth, assessed via occlusal relationships between the maxillary and mandibular arches on dental models taken at five years of age and analysed by the 5-Year-Old's occlusal index described by Attack et al.^15^ The 5-Year-Old's index has been accepted as the UK national audit tool at five years of age for the assessment of occlusal relationships in the primary dentition, prior to any orthodontic intervention.^16^

The 5-Year-Old's index assigns a score of 1 (good) to 5 (poor) on an ordinal scale, based on the relationship of upper and lower dental model casts. Each cleft centre sent their dental models to be assessed by two nationally calibrated orthodontists, who scored the models individually. Differences were resolved through discussion to reach a consensus. All orthodontic examiners underwent a calibration process via the UK Orthodontic Clinical Excellence Network, with inter- and intra-rater kappa scores above 0.8, in order to be accepted as a scorer in this process.

### Analysis

The relationship between UCLP sidedness and maxillary growth was analysed initially using descriptive statistics and cumulative percentages. These associations were quantified by Odds Ratio (OR, 95%CI) and *P* values.^17^ Due to current contention about the best way in which to analyse ordinal data,^18^ we took two regression approaches: First we calculated the OR of having a better outcome comparing left and right UCLP by logistic regression for each of the 4 potential cut points in the 5 -Year-Old's index ordinal data. Each cut point dichotomised the 5-Year-Old's index scores into better and worse outcome groups. Second, if the common odds assumption was demonstrated to be satisfied, we used a proportional odds logistic regression to estimate a common OR on sidedness effect. Adjustment was made for sex in each regression model in recognition of this potential explanatory variable in orofacial cleft sidedness research.^1^ Statistical analysis was performed using the R Project for Statistical Computing, version 4.3.1 (https://www.r-project.org)

## Results

There were 378 children included born with a complete UCLP, with data derived from seven UK cleft centres. 256 (68%) had a left sided UCLP and 122 (32%) had a right sided UCLP. 248 (66%) were male and 130 (34%) were female. Five Year Old's Index scores ranged from 1-5 with a mean score of 2.9 and a median score of 3.

**Figure 1.**
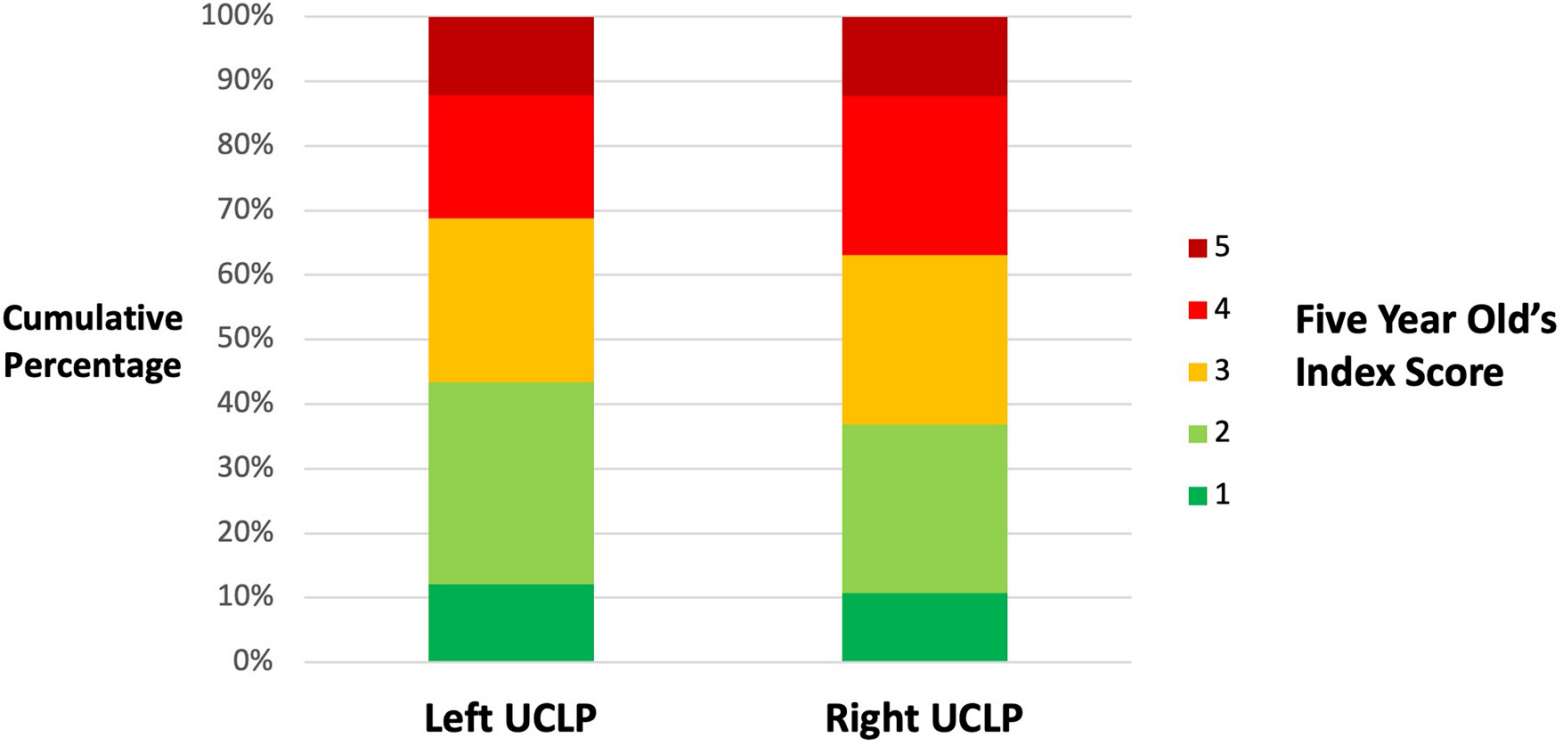
A cumulative bar chart to show the Five Year Old's Index scores (Ordinal scale indicating maxillary growth from 1 (good) to 5 (poor)) for left and right sided unilateral cleft lip and palate (UCLP).

Descriptive analysis (see Table 2 and Figure 1) shows a trend for slightly better maxillary growth in left sided UCLP, with a higher proportion of good 5-Year-Old's Index Scores (1 and 2) in left sided UCLP (43%) compared to right sided UCLP (37%). Effect estimates in all regression models show a consistent trend for better growth in left sided UCLP with odds of having better outcome in left UCLP constantly higher than in right UCLP group (all ORs > 1), yet there is weak evidence for a difference (as supported by the confidence intervals and *P* values) (see Table 3). The ORs comparing better to worse outcomes in left and right groups at different dichotomised cut points within the 5-Year-Old's Index showed that the common odds assumption was reasonable (OR range between 1.02 to 1.33). The summary effect estimate from the proportional odds model describes the odds of having better 5-Year-Old's Index Scores in left UCLP is 1.24 times higher compared to right UCLP having better 5-year-old's index, (OR 1.24, 95% CI 0.85 to 1.82; *P* = .27).

**Table 2.** 5-Year-Old's (5YO) index Scores for Left and Right UCLP and Females and Males. Frequencies and Cumulative Percentages Displayed.

5YO Index Score	Total	Left UCLP	Cumulative %	Right UCLP	Cumulative %	Female	Cumulative %	Male	Cumulative %
1	44	31	12.1%	13	10.7%	12	9.2%	32	12.9%
2	112	80	43.4%	32	36.9%	36	36.9%	76	43.5%
3	97	65	68.8%	32	63.1%	32	61.5%	65	69.8%
4	79	49	87.9%	30	87.7%	29	83.8%	50	89.9%
5	46	31	100.0%	15	100.0%	21	100.0%	25	100.0%
Total	378	256		122		130		248	

**Table 3.** Odds Ratios of Having Better 5-Year-Old's (5YO) Index Scores Comparing Left to Right Sided Unilateral Cleft lip and Palate With and Without Adjustment for Sex.

5YO Index analysis strategy	Unadjusted OR (95% Cis)	*P* value	Adjusted OR (95% Cis)	*P* value
Logistic regression: Dichotomised cut points				
1|2	1.16 (0.58 to 2.29)	.68	1.18 (0.59 to 2.34)	.64
2|3	1.31 (0.84 to 2.04)	.23	1.33 (0.85 to 2.07)	.21
3|4	1.29 (0.82 to 2.02)	.28	1.31 (0.83 to 2.07)	.24
4|5	1.02 (0.53 to 1.97)	.96	1.05 (0.54 to 2.03)	.89
Proportional odds model	1.24 (0.85 to 1.82)	.27	1.27 (0.87 to 1.87)	.22

For the covariable of sex, there was a trend for better maxillary growth (5-Year-Old's index scores 1 and 2) in males (43.5%) compared to females (36.9%), although there was weak evidence in a univariate proportional odds regression analysis to suggest a difference (OR 1.42, 95% CI 0.97 to 2.08; *P* = .07). Adjusting for sex in the sidedness regression models did not alter the trend of the unadjusted results (see Table 3).

## Discussion

### Summary of Key Findings

There was a trend for better maxillary growth in left compared to right sided UCLP at 5 years of age (OR 1.24, 95% CI 0.85 to 1.82; *P* = .27), yet the uncertainty around the effect estimates in the regression analysis of ordinal 5-Year-Old's index scores implies the evidence for a difference in growth between left and right sided UCLP is weak.

### Strength and Weaknesses

This study is strengthened by the sample of patients, which was homogeneous in terms of the complete UCLP phenotype, and the consistent measurement of exposure and outcome variables. The inclusion of multiple UK cleft centres increases the applicability of the findings, and whilst there is generalised adherence to the UK cleft surgery pathway,^19^ there is reported variability in surgical techniques, timings and sequences amongst UK surgeons,^20^ which are known to have an impact on maxillary growth. We adjusted for the co-variable of sex as this was readily available in our dataset but we were not able to add additional potentially important variables to our model, such as ethnicity.

The 5-Year-Old's Index is an important outcome as it has been accepted as the recognised national audit measure in the UK and has been recommended for use in primary dentition.^16^ The long-term predictive ability of the 5-Year-Old's index has been questioned, particularly of poor growth (scores 4 and 5 on the 5-Year-Old's index), which has shown poor correlation with growth outcomes at skeletal maturity.^21^

The analysis of ordinal data in healthcare research is notoriously challenging and there is a paucity of a gold standard in terms of analytic strategies.^18^ Dichotomising the ordinal scale is appealing for simplicity, yet the resulting effect estimate is only applicable for the arbitrarily chosen cut point and does not account for the variation of the data within each ordinal level.^22^ The use of the proportional odds logistic regression analysis maintains the integrity of the 5-Year-Old's index ordinal data and has been encouraged in healthcare outcome studies to retain clinically relevant information.^23^ The proportional odds model does rely on the assumption that the sidedness effect is constant across all cut points.^24^ This assumption was supported by our data with unadjusted ORs (Table 3) falling within a similar magnitude.

### Comparison to Other Studies

Whilst we are not aware of previous studies analyzing the relationship between UCLP sidedness and maxillary growth using the 5-Year-Old's index, other studies have investigated dental arch relationships using different outcome measures. Haque et al.^25^ in a case series from Bangladesh reported no difference in dental arch relationships between left and right UCLP at a mean age of eight years (before orthodontic or orthognathic intervention) using the GOSLON yardstick, dichotomised into a binary outcome of favourable versus unfavourable growth (OR 1.12, 95%CI 0.409 to 3.11; *P* = .83). Staudt et al.^26^ in a case series from Switzerland reported better post-treatment occlusion at a mean age of 19 years for left sided UCLP using Modified Huddart Bodenham scores, although the prevalence of orthognathic surgery was higher for left UCLP (65% vs 20%). An Iranian study investigating cephalometric measurement of facial growth at an unspecified age, reported no difference in most of the 28 measurements reported between left and right UCLP (including vertical and sagittal skeletal, dental and soft tissue measures), but within the four measurements noted to be different, there was greater mandibular length in left sided compared to right sided UCLP.^27^

### Interpretation and Further Work

The trend for better growth in left sided UCLP, reported in this study and previous studies, may suggest a subtle intrinsic influence of sidedness on maxillary growth outcomes. The severity of UCLP in terms of size of the alveolar cleft and circumference of the dental arch has been associated with maxillary projection.^11^ There is increasing evidence that sidedness may also be related to severity in the UCLP phenotype; right UCLP has been associated with a higher prevalence of additional congenital malformations,^28,29^ anatomical hypoplasia and asymmetries in the face.^2–4,30^ If patients born with right sided UCLP truly are at higher risk of maxillary growth disturbance, this has implications on patient counselling, and treatment options that might attempt to mitigate the increased risk.

The uncertainty around the effect estimate in this study may have a number of reasons. First, it is possible that UCLP sidedness does not impact maxillary growth. Second, the sample size may not have been sufficiently large enough to differentiate subtle differences in maxillary growth by sidedness. Third, the outcome measure at five years of age may not be the most appropriate to determine definitive growth outcome. Fourth, additional variables not considered within our model, both extrinsic (such as surgical technique) and intrinsic (such as ethnicity) may have an important influence. Further work is required to analyse maxillary growth in a large heterogenous sample of patients born with complete UCLP, ideally at skeletal maturity.

## Conclusion

We report a trend for better growth in left sided UCLP compared to right sided UCLP with a caveat that the strength of evidence for a difference is weak. Further work is warranted to investigate the impact of UCLP sidedness on outcomes including maxillary growth.
